# Proximal Margin Involvement Following Total Gastrectomy for Seiwert III Adenocarcinoma: A Management Dilemma

**DOI:** 10.7759/cureus.64945

**Published:** 2024-07-19

**Authors:** Rajdave S Sadu Singh, Guo H Loo, Guhan Muthkumaran, Sekkapan T Sambanthan, Nik Ritza Kosai

**Affiliations:** 1 Upper Gastrointestinal and Metabolic Surgery, Hospital Canselor Tuanku Muhriz, Universiti Kebangsaan Malaysia, Kuala Lumpur, MYS; 2 Surgery, Sultanah Aminah Hospital, Ministry of Health Malaysia, Johor Bharu, MYS

**Keywords:** colonic conduit, prehabilitation, poorly differentiated adenocarcinoma, junctional cancer, salvage surgery

## Abstract

Oesophagogastric junction carcinoma is now being increasingly regarded as a distinct site of neoplasia, separate from its adjacent sites. Recent advances in multimodal treatment approaches, including endoscopic procedures, oesophagectomy with three-field lymph node dissection, and definitive chemoradiotherapy, have significantly improved overall patient survival rates. Despite these advancements, the recurrence rate remains around 50% within one to three years following initial surgery. A major challenge in management arises when the resected surgical margins are involved with cancer.

We present a 55-year-old man who experienced progressive dysphagia and, upon further assessment, was noted to have a Siewert III oesophagogastric junction adenocarcinoma. He underwent neoadjuvant chemotherapy before undergoing total gastrectomy with D2 lymphadenectomy with a Roux-en-Y reconstruction. Histopathological examination of the resected specimen revealed a positive proximal margin involvement. After optimization, he then underwent a salvage three-field McKeown oesophagectomy with colonic conduit reconstruction and adjuvant chemotherapy.

Salvage surgery can be considered for patients with locoregional recurrence after definitive chemoradiotherapy or surgery. Other options include salvage chemoradiotherapy. Our case outlines the importance of proper patient selection for salvage surgery and highlights the choices of conduit in patients undergoing total esophagectomy post gastrectomy.

In conclusion, managing proximal margin involvement of cardioesophageal junction adenocarcinoma remains a complex and multifaceted challenge, necessitating a tailored, multidisciplinary approach. The decision-making process must consider the patient's functional status, previous treatments, and specific anatomical considerations.

## Introduction

Oesophagogastric junction (OGJ) carcinoma represents an anatomical site of neoplasia that is progressively being considered to arise autonomously from other neighboring sites [1]. The incidence of OGJ cancer has increased globally in recent years. In the United States, the number of patients has increased by four to five-fold over the past two decades. Squamous cell carcinoma (SCC) and adenocarcinoma are the two subtypes of this cancer, with an overall predominance of SCC; it accounts for 70% of cases worldwide [2].

OGJ adenocarcinomas are typically classified by the classification proposed by Siewert [3]. Siewert classified the lesion on the location of its epicenter: type I, the epicenter of lesion 1-5cm above OGJ; type 2, the epicenter located 1cm above or 2cm below OGJ; and type 3, the epicenter located 2-5cm below OGJ. The mainstay for curative treatment of OGJ cancer is multimodal and radical treatment [4]. However, an optimal surgical approach remains controversial due to the complex lymphatic drainage pathway of cancer into the abdomen and mediastinum. Here, we present a case highlighting the potential of salvage resection as a treatment option for patients with proximal margin involvement after initial surgery for a Siewert III OGJ adenocarcinoma.

## Case presentation

**Figure 1:**
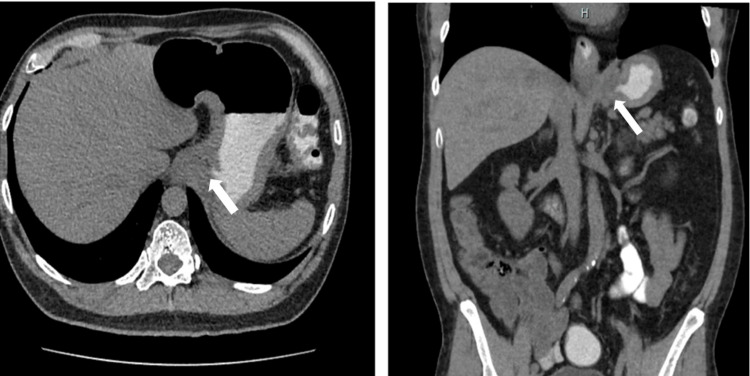
Contrast-enhanced CT of the abdomen axial section (left) and coronal section (right) showing circumferential concentric thickening extending from distal gastroesophageal junction till gastric cardia (white arrow)

A 55-year-old man presented with a six-month history of progressive dysphagia, although he remained able to tolerate a soft diet and liquids. During this period, he experienced an unintentional significant weight loss of 20kg. Subsequently, he sought medical attention at our center. An esophagogastroduodenoscopy (OGDS) and a contrast-enhanced computer tomography (CECT) of the thorax, abdomen, and pelvis revealed an esophagogastric tumor (Figure 1), classified as Siewert III, extending from the cardia to the proximal body of the stomach, no evidence of distant metastasis on CECT. Biopsy results indicated poorly differentiated adenocarcinoma. 

**Figure 2:**
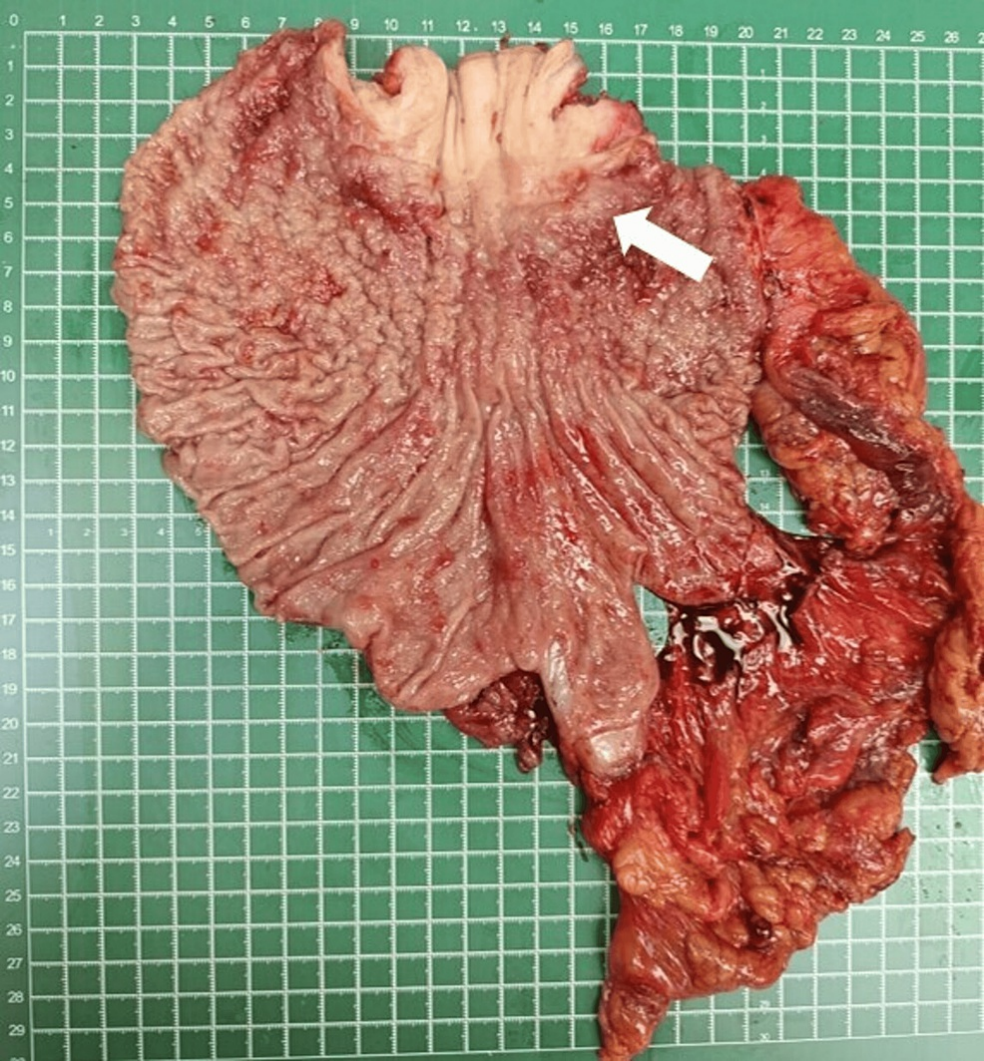
Total gastrectomy specimen, with the tumor seen at the esophagogastric junction (white arrow)

After a multidisciplinary team discussion, the patient underwent neoadjuvant chemotherapy (FLOT regime) and subsequently an open total gastrectomy, D2 lymphadenectomy, with a Roux-en-Y reconstruction (Figure 2). The postoperative period was uneventful, and he was discharged well after 10 days. The histopathological examination revealed poorly cohesive adenocarcinoma (non-signet cell type) classified as ypT4b N2, with involvement of the left crura and a positive proximal margin. There was also a presence of lymphovascular and perineural invasion, with three out of six nodes positive for nodal metastases. 

**Figure 3:**
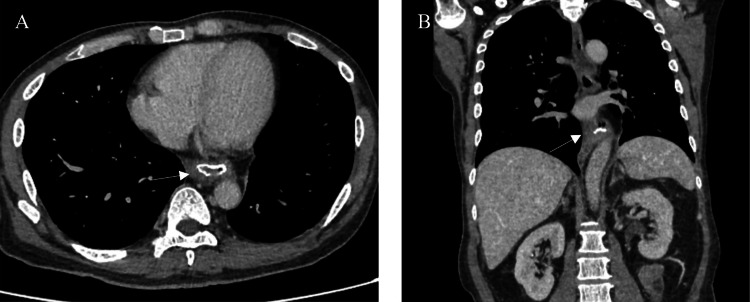
Contrast-enhanced CT of the thorax and abdomen in axial (A) and coronal (B) view showing enhancing thickening at esophagojejunostomy anastomotic site suggestive of possible local recurrence

A CECT restaging performed three weeks after index surgery showed minimally enhancing thickening at the esophagojejunostomy anastomotic site (Figure 3), suggesting tumor recurrence given the history of proximal margin involvement, although no distant metastasis was observed. An upper endoscopy performed showed no abnormal growth at the esophagojejunostomy site; hence, no biopsy was taken. A multidisciplinary team meeting, including radiologists, upper gastrointestinal surgeons, and oncologists, concluded that the best course of action would be salvage total esophagectomy followed by adjuvant chemotherapy.

**Figure 4:**
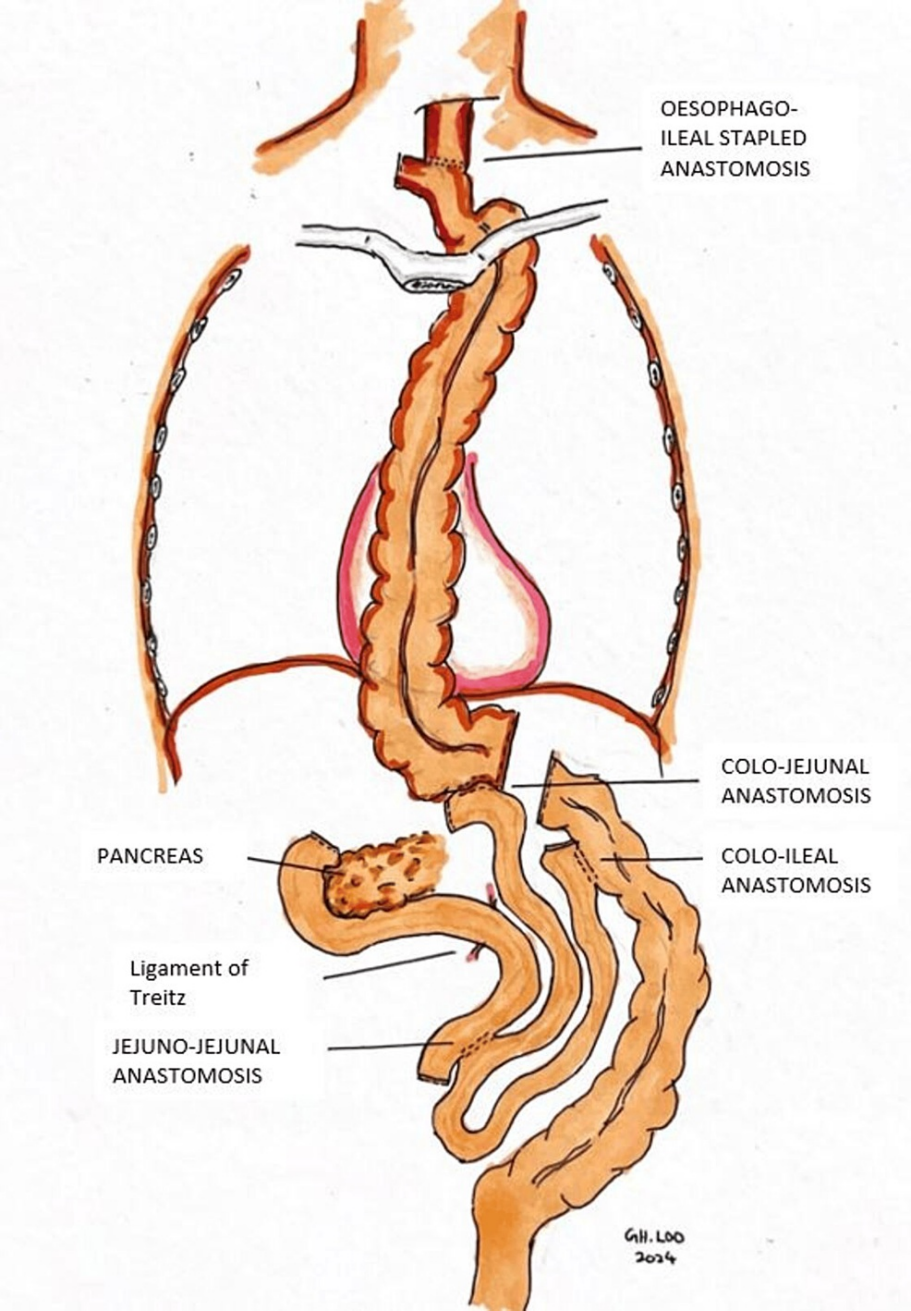
Illustration of esophagoileal-, colojejunal- and coloileal anastomosis performed, with colonic conduit seen within the thorax cavity

During preoperative planning, the patient underwent echocardiography, lung function tests, CT angiography mesentery (for conduit planning), and nutritional optimization as part of his prehabilitation, resulting in a weight gain of 3.5kg. He was then scheduled for a three-field McKeown esophagectomy with a right-sided colonic conduit. Intraoperatively, dense adhesions with neovascularization were observed at the previous anastomotic site, with the blind jejunal limb adhering densely to the left hemidiaphragm. The terminal ileum was used for anastomosis with the proximal remnant esophagus owing to the difficulty in bringing the caecum into the neck (Figure 4).

Postoperatively, the patient recovered well and was extubated on postoperative day four. He was started on total parenteral nutrition for seven days, followed by nasocolic jejunal tube feeding, receiving 35 kcal/kg/day. He developed left vocal cord neuropraxia (which recovered spontaneously). The patient was discharged on postoperative day 15 and is currently undergoing adjuvant chemotherapy. 

## Discussion

Involvement of the proximal margin after index surgery presents significant therapeutic challenges due to the disease's complexity and the often limited options available following initial treatment. Despite our patient being asymptomatic, computer tomography of the thorax, abdomen, and pelvis was repeated to ensure there wasn't any distant metastasis. A positron emission tomography-computed tomography (PET CT) scan was not performed due to the limited accurate interpretation due to 18F-fluorodeoxyglucose (FDG) avidity in post-surgical inflammatory uptake at the surgical anastomosis, the diminutive size of peritoneal metastases, and technical difficulty distinguishing vascular uptake from nodal uptake [5]. Other modalities of imaging, such as the MRI, have limited roles in patients post neoadjuvant and surgery, and the use of MRI alone would result in many complete responders being misdiagnosed as having residual disease and, therefore, a combination of modalities is needed to correctly diagnose complete responders when MRI used as a modality [6]. Hence, the ideal modality to restage our patient would be contrast-enhanced computer tomography of the thorax, abdomen, and pelvis with a complementary upper endoscopy assessment. 

In our patient, the likely reason for the progression of the disease was due to proximal margin involvement during the initial surgery (R1 resection). In hindsight, perhaps the use of an intraoperative frozen section in the index surgery would have avoided a second surgery. Despite this setback, a multidisciplinary team approach was undertaken, and salvage surgery was recommended. 

According to the Oesophageal Cancer Practice Guidelines 2022 by the Japan Esophageal Society (JES), salvage surgery can be considered for patients with locoregional recurrence after definitive chemoradiotherapy (CRT) or surgery [7]. However, this procedure is associated with substantial risks, including pulmonary complications and anastomotic leaks. A meta-analysis by Faiz et al. reported that the anastomotic leakage rate for salvage oesophageal surgery was 18.6%, pulmonary complications were 30.2 and the 90-day mortality rate was 8.8% [8]. Factors to consider when planning for surgery include the recurrence's location and extent, the patient's overall health, and previous treatments. The three-year overall survival (OS) ranges up to 78% for patients who underwent surgery for recurrence and have a positive circumferential margin [9].

Mckeown's three-field oesophagectomy was performed due to higher resection margins, extensive lymph node dissection, and reduced morbidity from thoracic complications in the event of an anastomotic leak. This makes it a preferred choice for proximal margin involvement in oesophageal cancer. These benefits lead to better oncologic outcomes and more manageable postoperative care [10]

For reconstruction after oesophagectomy, when a gastric conduit is unavailable due to previous gastrectomy, concurrent gastric cancer, or tumor invasion into the stomach, a colon conduit is preferable to a jejunal conduit. The colon provides a more extended graft, which is easier to bring up to the neck for anastomosis. Furthermore, the colon is a better conduit than the jejunum in terms of reservoir capacity, acid resistance, and reflux prevention. However, this complex surgical technique requires three anastomoses, significantly increasing the risk of anastomotic leak. The left colon is typically chosen between the left and right colon due to its extended size, thicker wall, smaller diameter, and almost universal presence of the left colic artery compared to the right colic artery, which is present in around 20% of the population [11]. In our patient, we proceeded with the right colonic conduit with terminal ileum attached to it due to the presence of the right colic artery. Yasuda et al. recommended enlarging the thoracic inlet by resecting muscle and tendons at the cervical base to prevent kinking of the graft when delivering the conduit substernal. Occasionally, the left portion of the manubrium, the head of the left clavicle, or even the left thyroid lobe can be resected to ensure no compression to the interposed graft [12].

Another option in this situation is salvage chemoradiotherapy (SCRT) [13]. While SCRT is recommended, particularly for patients who have not received prior radiotherapy, salvage surgery might offer superior oncological outcomes for certain patients with locoregional failure after CRT. However, the overall benefit of salvage surgery compared to other modalities remains a subject of ongoing research and debate [7].

In the Checkmate 577 trial, nivolumab was administered to all eligible patients regardless of their PD-L1 combined positive score (CPS). It was found that Nivolumab significantly extended disease-free survival compared to the placebo. Patients treated with nivolumab had a median DFS of 22.4 months compared to 11.0 months for those receiving the placebo [14]. Our patient histopathological examination of the esophagectomy revealed poorly differentiated adenocarcinoma with clear surgical margins; however, malignant cells were seen in the left and right diaphragmatic crus. Human epidermal growth factor receptor 2 was negative for this patient, and nivolumab was not offered to our patient due to financial limitations. He proceeded to complete the FLOT regime.

## Conclusions

Managing proximal margin involvement of OGJ tumours after definitive surgery remains a complex and multifaceted challenge, necessitating a tailored, multidisciplinary approach. The choice of reconstruction technique, mainly using a colon conduit, is critical in optimizing outcomes. Ongoing research and clinical guidelines continue to inform and refine these strategies, underscoring the importance of individualized treatment plans in improving survival and quality of life for patients with proximal margin involvement of OGJ tumours.
